# Unveiling the Hidden Responses: Metagenomic Insights into Dwarf Bamboo (*Fargesia denudata*) Rhizosphere under Drought and Nitrogen Challenges

**DOI:** 10.3390/ijms251910790

**Published:** 2024-10-08

**Authors:** Jun Xiang, Nannan Zhang, Jiangtao Li, Yue Zhu, Tingying Cao, Yanjie Wang

**Affiliations:** 1 College of Life Science, Sichuan Normal University, Chengdu 610101, China; 15196037910@163.com (J.X.); 15378658484@163.com (J.L.); 18121949467@163.com (Y.Z.); 17781029330@163.com (T.C.); 2 Chengdu Institute of Biology, Chinese Academy of Sciences, Chengdu 610213, China; zhangnn@cib.ac.cn

**Keywords:** dwarf bamboo, metagenomic sequencing, drought stress, nitrogen deposition

## Abstract

Dwarf bamboo (*Fargesia denudata*) is a crucial food source for the giant pandas. With its shallow root system and rapid growth, dwarf bamboo is highly sensitive to drought stress and nitrogen deposition, both major concerns of global climate change affecting plant growth and rhizosphere environments. However, few reports address the response mechanisms of the dwarf bamboo rhizosphere environment to these two factors. Therefore, this study investigated the effects of drought stress and nitrogen deposition on the physicochemical properties and microbial community composition of the arrow bamboo rhizosphere soil, using metagenomic sequencing to analyze functional genes involved in carbon and nitrogen cycles. Both drought stress and nitrogen deposition significantly altered the soil nutrient content, but their combination had no significant impact on these indicators. Nitrogen deposition increased the relative abundance of the microbial functional gene *nrfA*, while decreasing the abundances of *nirK*, *nosZ*, *norB*, and *nifH*. Drought stress inhibited the functional genes of key microbial enzymes involved in starch and sucrose metabolism, but promoted those involved in galactose metabolism, inositol phosphate metabolism, and hemicellulose degradation. NO_3_^−^-N showed the highest correlation with N-cycling functional genes (*p* < 0.01). Total C and total N had the greatest impact on the relative abundance of key enzyme functional genes involved in carbon degradation. This research provides theoretical and technical references for the sustainable management and conservation of dwarf bamboo forests in giant panda habitats under global climate change.

## 1. Introduction

In the context of global change, climate change factors do not operate in isolation; they occur simultaneously, leading to complex coupling effects. Studies have revealed significant variations in the impact on soil physicochemical properties, functions and microbial biodiversity when multiple climate change factors are combined [1,2]. This results in synergistic, antagonistic, or additive effects among these factors. Water availability and nitrogen, crucial factors for plant growth under climate change, are profoundly influenced. During drought stress, nitrogen deposition significantly alleviates the adverse impacts of drought stress on plants by increasing leaf PSII activity and leaf photosynthesis [3,4,5]. Additionally, anthropogenic nitrogen addition severely affects the ecosystem functioning of soil microbial communities in semi-arid temperate grasslands [6,7]. Despite these findings, there is a substantial gap in our understanding of the coupled effects of drought stress and nitrogen deposition on plants and their rhizospheric environment. Consequently, conducting further in-depth studies in this area is imperative.

Meanwhile, the intensification, frequency, and impact range of extreme weather and climate events, such as drought, are escalating due to global climate change [8]. Drought not only disrupts the coordinated development of the social economy but also degrades the ecological environment, leading to ecological security concerns [9]. Recent environmental pressures and global warming have caused arid regions to expand into humid areas in southern China, threatening nearly half of the total land area. Drought stress is thus emerging as a significant and urgent challenge for sustainable development in China [10]. Additionally, global atmospheric nitrogen deposition has seen a significant annual increase. With rapid economic development, China has become the world’s leading producer and emitter of reactive nitrogen, making it the primary nitrogen deposition region in Asia [11]. Consequently, the ecological impacts of both drought and nitrogen deposition, along with their intricate interconnections, have become hotspots in the global climate change discourse. Many researchers have delved into this topic, aiming to comprehend the complexities of these interactions [4,6,12]. Despite the progress, there is still much to explore to enhance our understanding of these intertwined phenomena and address the challenges they present.

The rhizosphere, located within a few millimeters from the root surface, differs from the surrounding soil in its physical, chemical, and biological properties. It serves as the primary gateway for nutrients or harmful substances to enter the plant [13] and acts as a vital nexus connecting plants, soil, and microorganisms. This microenvironment plays a crucial role in material cycling and capacity flow within ecosystems [14,15]. Rhizosphere changes are effective adaptations by plants to extreme environments, making it a significant domain for soil-root microbial interactions. Particularly in the context of global climate change, the rhizosphere has emerged as a focal point for research in plant physiology, soil science, microbiology, and ecology [16,17,18].

As a national treasure and the primary food source for giant pandas, dwarf bamboo (*Fargesia denudata*) mainly grows in the southwest alpine areas of China, offering essential ecological functions such as water conservation, soil stability, and nutrient balance. As a semi-woody plant with shallow roots, dwarf bamboo has a high water demand and is easily susceptible to drought conditions [19,20]. Drought can result in reduced bamboo shoots, shedding of bamboo leaves, and even premature flowering and death of dwarf bamboo [21]. Furthermore, due to its rapid growth, dwarf bamboo is also remarkably sensitive to nitrogen deposition [22]. Current research has explored the physiological responses to drought stress, nitrogen deposition, and their combined effects on bamboo [20]. However, there are limited studies on the response mechanisms of dwarf bamboo to drought stress and nitrogen deposition from the perspective of the rhizosphere environment. This research is vital in providing a scientific foundation and technical guidance for the management and protection of dwarf bamboo, which is essential for the habitat of giant pandas under future climate change.

This study focuses on the rhizosphere soil of dwarf bamboo, investigating the effects of drought stress and nitrogen deposition on the physicochemical properties, microbial community structure, and functional genes related to carbon and nitrogen cycling in the rhizosphere soil, utilizing techniques such as metagenomics. The aim is to further explore the mutual relationship between microbial and physicochemical properties in the rhizosphere soil of dwarf bamboo under the influence of drought stress and nitrogen deposition. The objective is to reveal the ecological adaptability mechanisms of the rhizosphere microenvironment of dwarf bamboo to drought stress and nitrogen deposition. This research provides a theoretical foundation and technical reference for the sustainable management and conservation of dwarf bamboo forests in the giant panda habitat under future climate change.

## 2. Results

### 2.1. Effects of Nitrogen (N) Deposition and Drought Stress on Soil Physicochemical Index

**Figure 1:**
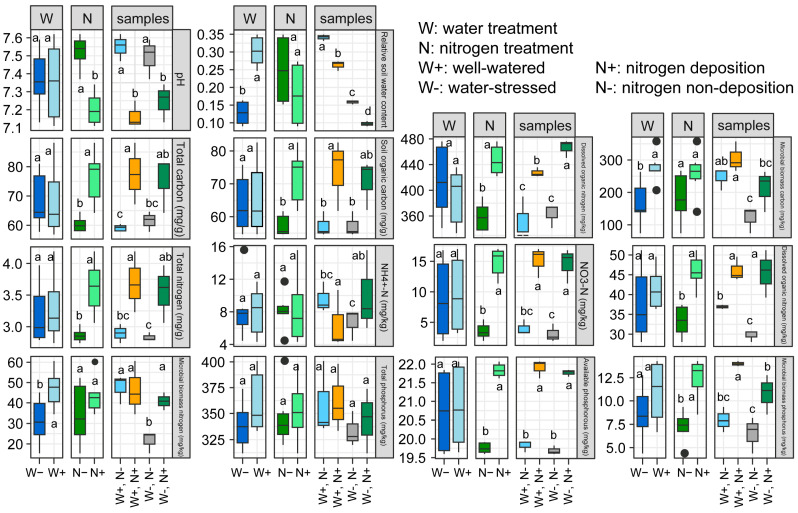
Effects of drought stress and nitrogen deposition on the rhizosphere soil physical-chemical properties of *Fargesia denudata* under non-nitrogen (N), deposition (−N), and N deposition (+N) with and without drought stress. Different letters indicate the significant differences between means, lower case indicates *p* < 0.05.

Soil nutrients are essential nutrients provided by the soil for plant growth. The dynamics of rhizosphere soil nutrients not only reflect the relationship between root-soil nutrient supply and demand but also indicate the influence of environmental conditions, exhibiting a high sensitivity to environmental changes [23]. Under both nitrogen deposition and non-deposition conditions, drought stress did not significantly affect the pH of rhizosphere soil (Figure 1). However, under well-watered and drought-stress conditions, nitrogen deposition significantly reduced soil pH. Regarding soil carbon, drought stress did not significantly alter the levels of total nitrogen (TN), soil organic carbon (SOC), and dissolved organic carbon (DOC) in the rhizosphere soil. Nevertheless, it notably decreased the microbial biomass carbon (MBC) content, while nitrogen deposition notably increased the concentrations of total carbon (TC), SOC, and DOC in the rhizosphere soil. Concerning soil nitrogen, drought stress showed no significant impact on the levels of total phosphorus (TP), ammonium nitrogen (NH_4_^+^-N), nitrate nitrogen (NO_3_^−^-N), and dissolved organic nitrogen (DON) in the rhizosphere soil, except for a significant reduction in microbial biomass nitrogen (MBN) content. As for soil phosphorus, nitrogen deposition markedly augmented the concentrations of available phosphorus (AP) and microbial biomass phosphorus (MBP) in the rhizosphere soil. However, no significant effects were observed on all phosphorus indices in the rhizosphere soil of dwarf bamboo under different watering conditions (Figure 1).

### 2.2. Effects of Nitrogen (N) Deposition and Drought Stress on Microbial Communities and Functions

After sequencing and read-processing, 3,348,077 effective reads were obtained with a total sequence length of 1,448,960,064 and an average sequence length of 431.49 bp (Table 1). Alpha diversity analysis showed that drought stress and nitrogen deposition did not significantly affect the Chao1 and ACE (*p* > 0.05), indicating that these treatments had no significant impact on the richness of the microbial community under rhizosphere soil (Table 2). However, both drought stress and nitrogen deposition significantly influenced the microbial community diversity, Shannon, and Simpson index, in rhizosphere soil (*p* < 0.05) (Table 2).

**Figure 2:**
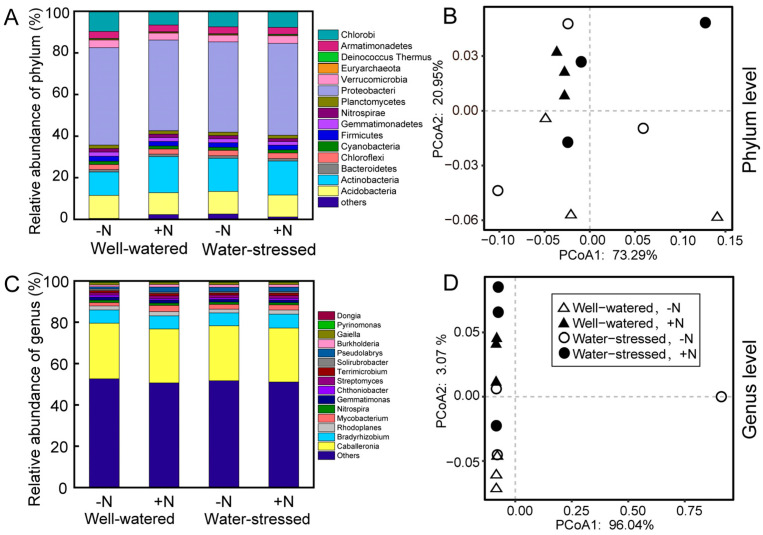
Relative abundance (**A**,**C**) and PCoA (**B**,**D**) of species at phylum and genus levels of the microbial community in rhizosphere soil of *Fargesia denudata* under different treatments.

The distribution and abundance of bacteria in soils at the phylum and genus levels were analyzed (Figure 2). On the phylum level, *Proteobacteria*, *Actinobacteria*, and *Acidobacteria* had the highest relative abundance among all samples (Figure 2A). Drought stress had no significant effect on the relative abundance of these phyla. While nitrogen deposition only significantly increased the relative abundance of *Actinobacteria*, increasing by 16.05% (Table S1). The analysis of PCoA and PERMANOVA also showed that both N deposition and drought stress, as well as their combination, have no effects on the microbial community structure in the rhizosphere soil at the phylum level (Table S2).

On the genus level, *Caballeronia*, *Bradyrhizobium*, *Rhodoplanes*, and *Mycobacterium* were the highest predominant genus among all samples (Figure 2C). Under normal water conditions, nitrogen deposition significantly increased the relative abundance of the genus *Bradyrhizobium* in the rhizosphere soil of dwarf bamboo, whereas nitrogen deposition increased the relative abundance of the genus *Rhizobium* under drought stress (Table S3). In the absence of N deposition, drought stress significantly increased the relative abundance of the genus *Bradyrhizobium*, whereas drought stress significantly increased the relative abundance of the genus *Rhizobium* under N deposition (Table S3).

Based on the KEGG pathway level-3 enrichment analysis, the relative abundance of microbial functional genes involved in nitrogen and carbon cycling was obtained (Figure S1). Most genes in N and C cycling were enriched in groups without N deposition, while the related genes decreased after N deposition. At the nitrogen metabolism level, we further analyzed the relative abundance changes of microbial functional genes involved in assimilatory nitrate reduction (*nirA*), dissimilatory nitrate reduction (*nrfA*), nitrification (*amoA*), denitrification (*narG*, *napA*, *nirK*, *nirS*, *norB* and *nosZ*), and nitrogen fixation (*nifH*) in the nitrogen cycle (Table 3). The relative abundance of functional genes related to N cycle, *nirK*, *nosZ*, *nifH* and *norB*, was significantly decreased after N deposition, while *nrfA* showed an upward trend in N deposition treatments. However, drought stress and nitrogen deposition did not significantly affect the relative abundance of functional genes, such as *nirA*, *nirS*, *narG*, *napA*, and *amoA* (Table 3).

At the carbon metabolism level, drought stress significantly affected the relative abundance of functional genes involved in inositol phosphate metabolism, whereas nitrogen deposition had a highly significant impact on the relative abundance of functional genes involved in C5-branched dibasic acid metabolism, citrate cycle, fructose, and mannose metabolism, glycolysis/gluconeogenesis, glyoxylate, and dicarboxylate metabolism, pentose and glucuronate interconversions, propanoate metabolism, pyruvate metabolism, carbon fixation in photosynthetic organisms, and carbon fixation pathways in prokaryotes (Table 4). The relative abundance of functional genes involved in galactose metabolism and starch and sucrose metabolism was markedly changed under the coupled nitrogen–drought stress treatment (Table 4).

At the carbon-degrading enzymes level, drought stress significantly affected the relative abundance of functional genes of hemicellulose-degrading enzymes (*p* < 0.05) (Table 5). Nitrogen deposition had a highly significant impact on the relative abundance of functional genes of cellobiose transporters, hemicellulose degradation, and sugar transporters (*p* < 0.05). The coupling effect of drought stress and nitrogen deposition did not significantly affect these functional genes involved in carbon degradation (*p* > 0.05) (Table 5).

### 2.3. Correlation between N Cycling-Related Microorganisms and Soil Physicochemical Index

**Figure 3:**
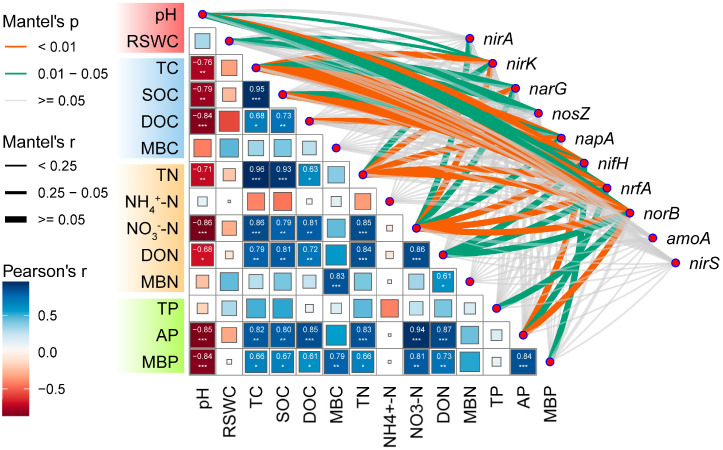
Correlation analysis of main microbial functional genes involved in nitrogen cycle with soil physical and chemical properties in rhizosphere soil of *Fargesia denudata.* *** *p* < 0.001; ** *p* < 0.01; * *p* < 0.05.

There was a correlation observed between the soil physicochemical properties and the relative abundance of microbial functional genes (Figure 3). These genes are involved in assimilatory nitrate reduction (*nirA*), dissimilatory nitrate reduction (*nrfA*), denitrification pathways (*narG*, *napA*, *nirK*, *nirS*, *norB*, *nosZ*), and nitrogen fixation pathways (*nifH*). However, no significant correlation was found with microbial functional genes involved in *amoA* (Figure 3). Among these genes, those involved in *nifH* exhibited the strongest correlation with the soil’s physicochemical properties (*p* < 0.01). Conversely, the correlation between the physicochemical properties and functional genes involved in *nirA* was the weakest, only demonstrating a significant negative correlation with NO_3_^−^-N (*p* < 0.05). Additionally, NO_3_^−^-N showed the highest correlation with functional genes associated with N-cycling (*p* < 0.01). Conversely, NH_4_^+^-N, MBC, and MBN exhibited no significant correlation with functional genes involved in N-cycling (*p* > 0.05).

### 2.4. Correlation between C Cycling-Related Microorganisms and Soil Physicochemical Index

**Figure 4:**
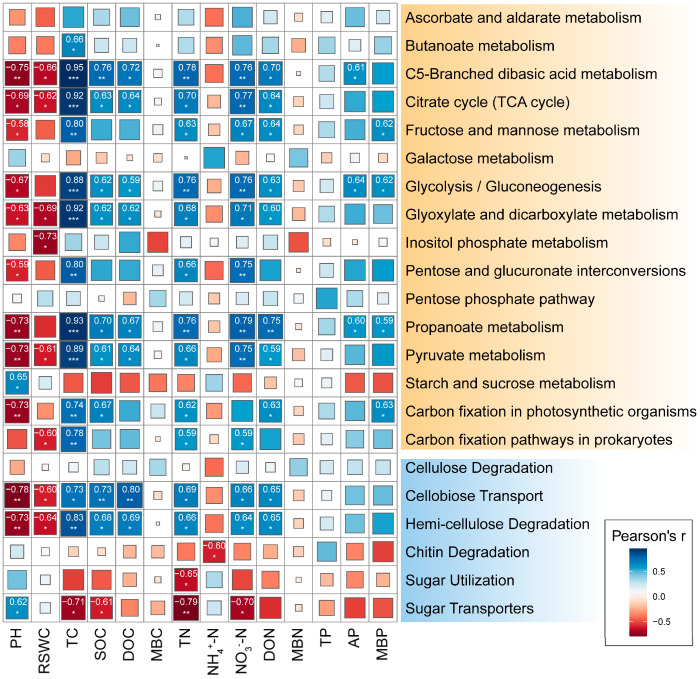
Correlation analysis of the main microbial functional genes involved in carbon cycle with soil physical and chemical properties in rhizosphere soil of *Fargesia denudata.* *** *p* < 0.001; ** *p* < 0.01; * *p* < 0.05.

The relative abundance of microbial functional genes involved in C5-branched dibasic acid metabolism, citrate cycle, glycolysis/gluconeogenesis, glyoxylate, and dicarboxylate metabolism, propanoate metabolism, pyruvate metabolism showed significant positive correlations with TC, SOC, DOC, TN, NO_3_^−^-N, and DON while showing negative correlations with pH and RSWC (Figure 4). However, MBC, MBN, and TP exhibited no significant correlation with the relative abundance of functional genes involved in C cycling. The relative abundance of the gene encoding for cellobiose transporter and hemicellulose degradation was significantly positively correlated with soil TC, SOC, DOC, TN, NO_3_^−^-N, and DON while showing a significant negative correlation with soil pH and RSWC. Cellulose degradation was not significantly correlated with soil physicochemical index. Chitin degradation and sugar utilization had a significant negative correlation with NH4+-N and TN, respectively. Sugar transporters were negatively correlated with four soil indices, including TC, SOC, TN, and NO_3_^−^-N. Overall, among the soil physicochemical indicators, TC and TN had the greatest impact on the relative abundance of key enzyme functional genes involved in carbon degradation.

## 3. Discussion

### 3.1. Response of Soil Physicochemical Index to Drought Stress and Nitrogen Deposition

Research on rhizosphere soil nutrients typically focuses on essential elements such as soil carbon, nitrogen, and phosphorus [24]. Drought stress and nitrogen deposition are significant environmental factors driving changes in rhizosphere soil carbon, nitrogen, and phosphorus [25]. Drought can alter soil nutrient dynamics, leading to reduced effectiveness [26]. Our study demonstrated that drought stress decreases MBC, MBN, and MBP in rhizosphere soil. This decline may be due to increased nutrient uptake by drought-stressed dwarf bamboo or reduced nutrient transformations. While appropriate N deposition can enhance N mineralization and organic matter decomposition, excessive N can disrupt soil nutrient balance and lead to acidification [27,28]. Our findings revealed that N deposition significantly increased TC, SOC, DOC, TN, NO_3_^−^-N, DON, AP, and MBP levels in dwarf bamboo rhizosphere soil, promoting nutrient accumulation (Figure 1) [27]. Additionally, N deposition may enhance available N content and photosynthetic assimilation, improving drought resistance [29]. These results suggest that N deposition enhances rhizosphere soil nutrient content in drought-stressed dwarf bamboo, augmenting its adaptation to dry conditions.

### 3.2. Response of Soil Microbial Community Structure and Diversity to Drought Stress and Nitrogen Deposition

Soil microbial community structure and diversity serve as important indicators of soil quality, being highly sensitive to external environmental conditions [30]. Studies have found that soil properties, vegetation types, and climatic conditions significantly influence soil microbial diversity [31,32]. Here, metagenomic sequencing technology was used to investigate the microbial community structure and diversity in the rhizosphere of dwarf bamboo under drought stress and nitrogen deposition. Results revealed that *Proteobacteria*, *Actinobacteria*, and *Acidobacteria* were the predominant phyla (Figure 2), which is consistent with the previous findings [33,34]. Drought stress and nitrogen deposition significantly increased the relative abundance of the genus *Bradyrhizobium* in the rhizosphere soil of arrow bamboo (Table S3). *Bradyrhizobium*, belonging to the phylum Proteobacteria, is known for nitrogen fixation and phosphorus solubilization, benefiting plant growth [35,36]. Thus, nitrogen deposition and its coupling with drought stress enhance nitrogen fixation and phosphorus solubilization abilities of rhizosphere soil microbes, promoting nutrient absorption and utilization by arrow bamboo, consequently enhancing its growth. Previous studies have found that water and nitrogen significantly alter soil microbial community structure, but their coupling mostly exhibits antagonistic effects [37,38]. Similarly, our study also demonstrates that both drought stress and nitrogen deposition affected soil microbial community diversity in the dwarf bamboo rhizosphere, with their combination also showing antagonistic effects. This means that the combined effect of both stressors did not amplify changes in microbial community structure as might be expected, but rather dampened or neutralized each other’s influence in certain microbial populations. This could be due to the fact that nitrogen deposition may alleviate some of the limitations caused by drought stress, or that drought conditions might restrict the effectiveness of nitrogen’s influence on microbial activities. These antagonistic interactions have important implications for understanding how complex environmental factors simultaneously influence soil microbial dynamics and plant-microbe interactions, particularly in changing climate conditions.

### 3.3. The Effect of N Deposition on Microbial Functional Genes for Soil C-N Cycling

As major climate factors of global climate change, drought stress, and nitrogen deposition can alter soil carbon and nitrogen transformation processes [39]. Many scholars have conducted research on the effects of drought stress on microbial functional genes involved in nitrogen cycling [40,41,42]. However, these studies typically focused on individual environmental factors. With the exacerbation of global climate change, multiple environmental factors often occur simultaneously or coexist. Therefore, we investigated the impacts of drought and nitrogen deposition on soil microbial functional genes involved in nitrogen cycling in the rhizosphere soil of arrow bamboo. We found that drought stress alone or coupled with nitrogen deposition had little effect on most microbial functional genes involved in nitrogen cycling, while nitrogen deposition significantly influenced many of these genes (Table 3). Specifically, nitrogen deposition markedly increased the relative abundance of the functional gene *nrfA* involved in dissimilatory nitrate reduction, while decreasing the relative abundance of *nirK*, *nosZ*, and *norB* genes involved in denitrification (Table 3). This indicates that nitrogen deposition suppresses denitrification, reducing nitrogen loss and accelerating the conversion of NO_2_^−^ to NH_4_^+^ in the rhizosphere soil [43]. Consequently, it was shown to enhance nitrogen utilization by arrow bamboo. The observed increase in *nrfA* and decrease in *nirK* genes are notable, as they highlight specific shifts in nitrogen cycling pathways under nitrogen deposition. *nrfA* encodes for a key enzyme in the dissimilatory nitrate reduction to ammonium (DNRA) pathway, while *nirK* is involved in the denitrification process, converting nitrite (NO_2−_) to nitric oxide (NO) [44]. The increase in *nrfA* suggests that nitrogen deposition enhances DNRA activity, leading to a greater retention of nitrogen in the form of ammonium (NH_4_^+^), which is more readily available for plant uptake. In contrast, the decrease in *nirK* indicates a suppression of denitrification, which reduces nitrogen loss from the system in the form of nitrogen gases (e.g., N_2_, N_2_O).

These shifts in microbial nitrogen cycling pathways can have significant ecological implications for the overall nitrogen budget of the ecosystem. The increase in DNRA activity promotes nitrogen conservation within the soil, as ammonium is retained rather than lost to the atmosphere through denitrification [45]. This can lead to a more efficient nitrogen cycle, where plants such as arrow bamboo benefit from enhanced nitrogen availability. However, the reduction in denitrification could alter the balance of nitrogen gases emitted from the soil, potentially decreasing the release of greenhouse gases such as nitrous oxide (N_2_O), a potent greenhouse gas [46]. Furthermore, the shift from denitrification to DNRA may influence the microbial community composition and soil chemical properties over time [47]. With more ammonium being retained in the soil, this could lead to increased ammonium toxicity or soil acidification if nitrogen deposition continues over the long term, potentially affecting soil health and plant-microbe interactions. However, nitrogen deposition had no significant effect on the relative abundance of functional genes involved in nitrification (Table 3), suggesting that nitrogen deposition may not consistently promote nitrogen cycling in the rhizosphere soil of arrow bamboo. This could be due to soil acidification and reduced microbial activity upon reaching certain nitrogen levels, leading to inhibition of nitrogen cycling [48,49]. Nitrogen deposition application reduced the activity and diversity of nitrogen-fixing microbes, inhibiting their nitrogen-fixing function [50,51,52]. Similarly, our study found that nitrogen deposition significantly decreased the relative abundance of the nitrogen fixation gene (*nifH*), suggesting that nitrogen deposition may reduce the activity of nitrogen-fixing microbes.

In the process of carbon cycling, both drought stress and nitrogen deposition can affect the soil microbes or their major functional genes involved in carbon cycling. Li, Xie [53] and Kuerban, Cong [54] found that nitrogen and water treatments altered the functionality of soil microbes, leading to changes in microbial metabolic activity and utilization of carbon sources. Some studies have also suggested that nitrogen deposition may stimulate the expression of microbial functional genes involved in carbon degradation, thereby increasing the decomposition of starch, chitin, and pectin in the soil [55,56,57]. This study also found that drought stress inhibited the relative abundance of functional genes of carbon cycling (cellulose-degrading enzymes), while nitrogen deposition significantly promoted the relative abundance of most functional genes involved in carbon cycling (cellulose transporter and cellulose-degrading enzymes) (Table 5). However, the coupling of these functional genes showed no significant effect. This indicated that drought stress inhibits the decomposition rate of organic matter, while nitrogen deposition promotes organic matter decomposition, which is consistent with the findings in the grasslands under nitrogen deposition conditions [7,58]. On a broader scale, these changes in carbon metabolism can significantly influence soil nutrient cycling. The increased microbial activity related to cellulose degradation and lignin decomposition under nitrogen deposition can enhance organic matter turnover, releasing carbon compounds that contribute to soil organic matter formation. This process improves carbon sequestration and helps maintain soil structure and fertility. Additionally, enhanced carbon cycling indirectly affects other nutrient cycles, particularly nitrogen and phosphorus, as microbes release and recycle these nutrients during organic matter decomposition. Thus, nitrogen deposition not only increases carbon degradation but also promotes nutrient availability for plant uptake, ultimately supporting ecosystem productivity. However, the suppression of microbial activity under drought conditions limits these processes, reducing nutrient cycling efficiency. However, there is still a limited and incomplete understanding of the effects of drought and nitrogen deposition on functional genes involved in carbon cycling in forest rhizosphere soil, necessitating further research in this area.

The combined effects of drought and nitrogen deposition were not as significant as anticipated. One possible explanation is that the microbial community may exhibit compensatory mechanisms, where nitrogen deposition might alleviate some of the limitations imposed by drought stress, or drought could limit the impact of nitrogen deposition. Additionally, the duration of the stress treatments in this study was relatively short so a longer duration might lead to more pronounced effects. Finally, soil characteristics such as moisture retention and pH could have played a role in modulating microbial responses, thus limiting the observable effects of the combined stresses. Future studies should consider longer stress durations and more variable environmental conditions to gain a better understanding of the interactions between these factors.

### 3.4. The Effect of Soil Physicochemical Index on Microbial Communities

Drought stress and nitrogen deposition have led to changes in soil physicochemical properties and nutrient distribution, which affect the diversity, structure, and function of soil microbial communities [59,60]. Therefore, we conducted the correlation analysis between the soil physicochemical properties and its microbial community to reveal the response of soil physicochemical properties and microbial communities to drought stress and nitrogen deposition. Previous studies have demonstrated that soil pH and nutrients are significant driving factors in the variation of soil microbial community structure [61,62]. Similarly, in this study a significant correlation was found between pH and nutrients and the functional genes involved in carbon and nitrogen cycling, highlighting soil pH and nutrients as crucial factors affecting the structure and function of soil microbial communities in *Fargesia* (Figure 3 and Figure 4). The concentration of NO_3_^−^-N is the main factor influencing microbial functional genes involved in nitrogen cycling, whereas total carbon, total nitrogen, and nitrate nitrogen content are the main factors influencing microbial functional genes involved in carbon cycling. We speculate that nitrogen deposition might primarily affect the microbial communities involved in soil carbon and nitrogen cycling by increasing the content of total carbon, total nitrogen, and nitrate nitrogen, while drought stress had no significant effect on the soil nutrient content, thus exerting a relatively smaller impact on the microbial communities involved in soil carbon and nitrogen cycling.

However, the combined effects of drought and nitrogen deposition were not as significant as anticipated. One possible explanation is that the microbial community may exhibit compensatory mechanisms, where nitrogen deposition might alleviate some of the limitations imposed by drought stress, or drought could limit the impact of nitrogen deposition. Additionally, the duration of the stress treatments in this study was relatively short, and a longer duration might lead to more pronounced effects. Finally, soil characteristics such as moisture retention and pH could have played a role in modulating microbial responses, limiting the observable effects of the combined stresses. Future studies should consider longer stress durations and more variable environmental conditions to gain a better understanding of the interactions between these factors.

## 4. Materials and Methods

### 4.1. Research Area and Experiment Design

The experiment site was located at the Maoxian Ecological Research Station, Chinese Academy of Sciences (103°53′58″ E, 31°41′07″ N, 1820 ma.s.l.) in southwestern China. The healthy and uniform dwarf bamboo plants (2 years old) were collected from the nursery at Wangliang National Nature Reserve (103°55′ E, 32°49′ N) and transplanted into 50 L plastic pots with 70 kg of homogenized soil. One standard plant with 4–5 ramets was cultured in each pot. All plants were grown in a semi-controlled solar greenhouse with ambient conditions of 8–33 °C, relative humidity of 40–85%, and watered every 3 days.

Seven months after transplantation, the experimental design was applied with two factors (water regime and N deposition). The N deposition treatments were performed, including without N deposition [0 g N m^−2^ year^−1^ (−N): no addition of NH_4_NO_3_ solutions to each pot] and with N deposition [10 g N m^−2^ year^−1^ (+N), addition of 200 mL NH_4_NO_3_ solutions (6.18 mM N) to each pot weekly]. The amount of N addition level was determined according to the atmospheric N accumulation rate in the study area (3.9 g N m^−2^ year^−1^) [63], this increased level was used to simulate elevated nitrogen deposition levels commonly observed in rapidly developing industrial regions. After two months of N deposition treatments, different water treatments were performed. Well-watered (W+): soil was maintained at 80–85% relative soil water content (RSWC), simulating conditions of adequate water availability. Water-stressed (W−): soil was maintained at 30–35% RSWC, simulating drought conditions by withholding water for 30 days. The chosen RSWC levels for well-watered and water-stressed treatments were based on prior studies that have identified these thresholds as critical for plant physiological responses under drought conditions in similar environments [64,65]. RSWC in each treatment was controlled by the weight method, where soil moisture was adjusted accordingly to maintain target levels throughout the experiment. Each treatment had three replications with nine standard plants per replication. At the end of the experiment, the rhizosphere soil was collected from the plant and placed into plastic bags for metagenome analysis and the physiochemistry properties. Air-dried soil samples were filtered through a 1-mm sieve.

### 4.2. Soil Physiochemistry Properties

To characterize the soil under different treatments, we analyzed 13 soil physical-chemical properties. Soil pH was measured in soil suspension with distilled water (1:2.5 *w*/*v*) using a pH meter (FE20, Mettler Toledo, Shanghai, China). Total carbon (TC) and total nitrogen (TN) of the soil were measured using a Vario MACRO cube Elemental analyzer (Elementar, Hanau, Germany). Dissolved organic carbon (DOC), soil organic carbon (SOC), and dissolved organic nitrogen (DON) were measured using a total carbon analyzer (Multi N/C 2100, Jena Analytic, Langewiesen, Germany). The concentrations of NH_4_^+^-N and NO_3_^−^-N were determined using SEAL AA3 continuous flow analysis (SEAL Analytical, Norderstedt, Germany). The total phosphorus (TP) of the soil was determined using H_2_SO_4_-HClO_4_ digestion. Available phosphorous (AP) was determined by the molybdenum-antimony-scandium colorimetry method. Soil microbial biomass phosphorus (MBP), microbial biomass carbon (MBC), and microbial biomass nitrogen (MBN) were measured using the chloroform fumigation extraction method. 

### 4.3. Metagenome Sequencing

One gram of the soil sample was used for microbial DNA extraction (Power Soil DNA Isolation Kit, Qiagen, Hilden, Germany) according to the manufacturer’s instructions. The extracted DNA was detected by 2% agarose gel electrophoresis, and the purity of DNA was quantitatively analyzed with NanoDrop 2000 (Thermo Scientific, Wilmington, NC, USA). To construct the paired-end library, 300 bp DNA fragments were randomly sheared using Covaris M220 (Covaris Inc., Woburn, MA, USA) and end-repair, dA-tailing, and adapter ligation procedures were performed using NEBNext Rapid DNA-Seq (Bioo Scientific, Austin, TX, USA). Sequencing was performed on an Illumina Hiseq Xten (Illumina Inc., San Diego, CA, USA) at Majorbio Biotech Co., Ltd. (Shanghai, China).

Illumina raw reads were cleaned and trimmed using fastp v0.23.2 to remove low-quality reads [66]. Clean data were then assembled to obtain contigs based on the succinct de Bruijn graph method by MEGAHIT software (version 1.2.9), and then the contigs were used to predicate the open reading frames (ORFs) using MATAGENE [67]. Then, the predicted ORFs (>100 bp) were translated to protein sequences and clustered at 95% sequence identity and 90% coverage using CD-HIT v4.6 (http://www.bioinformatics.org/cd-hit/ (accessed on 10 September 2019)), and the longest genes of every cluster were selected as the representative sequence to construct a nonredundant gene catalog. The high quality of each sample read was blasted with a nonredundant gene set at 95% sequence identity to obtain the abundance of genes in each sample using SOAPaligner software (version 2.21). Gene taxonomic classification and functional annotation were performed against the NCBI-NR database, COG (Clusters of orthologous and Groups), and KEGG (Kyoto Encyclopedia of Genes and Genomes) databases, respectively, using a BLASTP search (BLAST Version 2.2.28+) [68].

### 4.4. Statistical Analysis

All data were analyzed by one-way analysis of variance (ANOVA), and multiple comparisons among treatments were performed using the least significant difference (LSD) method. Then, two-way ANOVA was used to evaluate the effects of water regime, nitrogen deposition, and their interaction on all dependent variables. Principal Coordinates Analysis (PCoA) based on Euclidean distance was used to assess the microbial community structure in the rhizosphere soil. Finally, Pearson correlation analysis was applied to evaluate the correlation between rhizosphere soil physicochemical properties and microbial community. All data analyses were performed using Excel 2016, SPSS 24, and R 2.5.6 software. Graphs were generated using Origin 9.0 software and packages such as vegan and ggplot2 in R 2.5.6 were utilized. The raw data from metagenome sequencing was deposited in NCBI under the accession number PRJNA1120641.

## 5. Conclusions

This study highlights the significant impacts of drought stress and nitrogen deposition on the rhizosphere environment of dwarf bamboo (*Fargesia denudata*), a vital food source for giant pandas. This research demonstrates that both factors alter soil nutrient content and microbial community composition, although their combined effect is insignificant. Specifically, nitrogen deposition increases the relative abundance of the microbial functional gene *nrfA* and decreases the abundances of *nirK*, *nosZ*, *norB*, and *nifH*, indicating changes in nitrogen cycling processes. Drought stress influences microbial functional genes differently, inhibiting those related to starch and sucrose metabolism while promoting genes involved in galactose metabolism, inositol phosphate metabolism, and hemicellulose degradation. Additionally, this study identifies NO_3_^−^-N as highly correlated with N-cycling functional genes and finds that total C and total N significantly impact the relative abundance of key enzyme functional genes involved in carbon degradation. These findings enhance our understanding of the response of microbial functional genes involved in the soil carbon and nitrogen cycles to drought stress and nitrogen deposition, allowing for better predictions of the structure and function of soil microbial communities under global change.

## Figures and Tables

**Table 1:** Metagenome sequencing data for different treatments.

Treatments	Raw Reads	Clean Reads	Clean Per	Contigs	Bases (bp)	N50 (bp) N90 (bp)		Max (bp) Min (bp)	
Well-watered, −N	99,089,384	97,764,518	98.66%	601,083	326,882,166	557	337	20,153	300
	83,740,618	82,770,650	98.84%	432,627	225,993,005	532	335	8520	300
	93,404,480	92,337,966	98.86%	596,467	319,518,642	547	335	43,654	300
Well-watered, +N	95,058,150	94,005,278	98.90%	468,145	232,774,876	501	332	7784	300
	97,124,186	96,005,208	98.85%	484,206	240,004,615	499	332	6819	300
	96,808,852	95,707,126	98.86%	489,179	240,032,836	497	332	8035	300
Water-stressed, −N	92,819,990	101,910,784	98.85%	507,764	249,379,261	492	330	9944	300
	94,154,460	931,12,574	98.90%	520,176	268,941,072	522	334	10,380	300
	103,093,822	91,759,092	98.86%	553,203	289,580,691	531	335	13,386	300
Water-stressed, +N	92,969,798	91,811,334	98.75%	451,016	222,705,758	500	331	5748	300
	93,749,160	94,866,738	98.81%	549,649	286,257,660	528	335	7542	300
	96,007,016	92,545,234	98.72%	440,843	216,002,776	496	331	6582	300

**Table 2:** Diversity index in rhizosphere soil of *Fargesia* under different treatments.

Diversity Index	Well-Watered		Water-Stressed		W	N	W × N
	−N	+N	−N	+N			
Shannon	6.74 ± 0.02 ab	6.75 ± 0.02 a	6.76 ± 0.01 a	6.37 ± 0.02 b	ns	ns	*
Simpson	0.99 ± 0.00 b	0.99 ± 0.00 a	0.99 ± 0.00 ab	0.99 ± 0.00 ab	ns	ns	*
Chao 1	11,827.66 ± 29.48 a	11,794.67 ± 49.17 a	11,858 ± 39.94 a	11,728 ± 53.11 a	ns	ns	ns
ACE	11,827.67 ± 29.48 a	11,794.67 ± 49.17 a	11,858 ± 39.94 a	11,728 ± 53.11 a	ns	ns	ns

Note: different letters represent significant differences among the treatments. * *p* < 0.05; ns: There was no significant difference (*p* > 0.05).

**Table 3:** Relative abundance of main microbial functional genes involved in the nitrogen cycle in rhizosphere soil of *Fargesia denudata* under different treatments.

Genes	Well-Watered		Water-Stressed		W	N	W × N
	−N	+N	−N	+N			
*nirA*	1.87 × 10^−4^ a	1.60 × 10^−4^ a	1.72 × 10^−4^ a	1.45 × 10^−4^ a	ns	ns	ns
*nirK*	1.88 × 10^−4^ a	1.52 × 10^−4^ b	1.65 × 10^−4^ ab	1.45 × 10^−4^ b	ns	*	ns
*nirS*	4.34 × 10^−6^ a	2.89 × 10^−6^ a	2.75 × 10^−6^ a	2.31 × 10^−6^ a	ns	ns	ns
*narG*	1.95 × 10^−4^ a	1.56 × 10^−4^ a	1.74 × 10^−4^ a	1.53 × 10^−4^ a	ns	ns	ns
*nosZ*	4.34 × 10^−5^ a	2.86 × 10^−5^ b	3.09 × 10^−5^ b	2.98 × 10^−5^ b	ns	*	*
*napA*	1.53 × 10^−4^ a	1.20 × 10^−4^ a	1.36 × 10^−4^ a	1.24 × 10^−4^ a	ns	ns	ns
*nifH*	4.77 × 10^−6^ a	2.31 × 10^−6^ b	5.35 × 10^−6^ a	2.02 × 10^−6^ b	ns	*	ns
*norB*	2.00 × 10^−4^ a	1.30 × 10^−4^ b	1.75 × 10^−4^ ab	1.36 × 10^−4^ b	ns	*	ns
*amoA*	1.69 × 10^−5^ a	1.40 × 10^−5^ a	1.46 × 10^−5^ a	1.23 × 10^−5^ a	ns	ns	ns
*nrfA*	1.01 × 10^−4^ b	7.91 × 10^−5^ a	1.03 × 10^−4^ b	7.69 × 10^−5^ a	ns	*	ns

Note: different letters represent significant differences among the treatments (*p* < 0.05). * *p* < 0.05; ns: There was no significant difference (*p* > 0.05).

**Table 4:** Relative abundance of functional genes involved in carbon metabolism in rhizosphere soil of *Fargesia denudata* under different treatments.

Microbial Functional Genes Involved in the Carbon Cycle	Well-Watered		Water-Stressed		W	N	W × N
	−N	+N	−N	+N			
Butanoate metabolism	2.28 × 10^−2^ a	2.32 × 10^−2^ a	2.31 × 10^−2^ a	2.31 × 10^−2^ a	ns	ns	ns
C5-Branched dibasic acid metabolism	7.94 × 10^−3^ b	8.40 × 10^−3^ a	8.17 × 10^−3^ ab	8.43 × 10^−3^ a	ns	**	ns
Citrate cycle	2.21 × 10^−2^ b	2.37 × 10^−2^ a	2.29 × 10^−2^ ab	2.35 × 10^−2^ a	ns	**	ns
Fructose and mannose metabolism	7.83 × 10^−3^ b	8.16 × 10^−3^ a	7.96 × 10^−3^ ab	8.18 × 10^−3^ a	ns	*	ns
Galactose metabolism	6.26 × 10^−3^ a	6.16 × 10^−3^ b	6.19 × 10^−3^ ab	6.27 × 10^−3^ a	ns	ns	*
Glycolysis/Gluconeogenesis	2.63 × 10^−2^ b	2.71 × 10^−2^ a	2.67 × 10^−2^ ab	2.71 × 10^−2^ a	ns	**	ns
Glyoxylate and dicarboxylate metabolism	2.81 × 10^−2^ b	2.99 × 10^−2^ a	2.91 × 10^−2^ ab	3.03 × 10^−2^ a	ns	*	ns
Inositol phosphate metabolism	3.59 × 10^−3^ b	3.61 × 10^−3^ b	3.66 × 10^−3^ b	3.83 × 10^−3^ a	*	ns	ns
Pentose and glucuronate interconversions	3.82 × 10^−3^ b	4.09 × 10^−3^ a	3.97 × 10^−3^ ab	4.09 × 10^−3^ a	ns	*	ns
Pentose phosphate pathway	1.78 × 10^−2^ a	1.80 × 10^−2^ a	1.78 × 10^−2^ a	1.77 × 10^−2^ a	ns	ns	ns
Propanoate metabolism	2.13 × 10^−2^ b	2.25 × 10^−2^ a	2.19 × 10^−2^ ab	2.25 × 10^−2^ a	ns	*	ns
Pyruvate metabolism	3.07 × 10^−2^ b	3.21 × 10^−2^ a	3.14 × 10^−2^ ab	3.20 × 10^−2^ a	ns	**	ns
Starch and sucrose metabolism	1.62 × 10^−2^ a	1.56 × 10^−2^ b	1.57 × 10^−2^ b	1.58 × 10^−2^ ab	ns	n	*
Carbon fixation in photosynthetic organisms	1.07 × 10^−2^ b	1.12 × 10^−2^ a	1.10 × 10^−2^ ab	1.10 × 10^−2^ ab	ns	*	ns
Carbon fixation pathways in prokaryotes	3.29 × 10^−2^ b	3.37 × 10^−2^ ab	3.33 × 10^−2^ ab	3.38 × 10^−2^ a	ns	*	ns

Note: different letters represent significant differences among the treatments (*p* < 0.05). ** *p* < 0.01; * *p* < 0.05; ns: There was no significant difference (*p* > 0.05).

**Table 5:** Relative abundance of functional genes involved in carbon degradation in rhizosphere soil of *Fargesia denudata* under different treatments.

Microbial Functional Genes Involved in Carbon Degrading Enzymes	Well-Watered		Water-Stressed		W	N	W × N
	−N	+N	−N	+N			
Cellulose Degradation	1.56 × 10^−3^ a	1.58 × 10^−3^ a	1.54 × 10^−3^ a	1.58 × 10^−3^ a	ns	ns	ns
Cellobiose Transport	2.04 × 10^−5^ c	4.13 × 10^−5^ ab	3.19 × 10^−5^ cb	5.06 × 10^−5^ a	ns	**	ns
Hemi-cellulose Degradation	2.16 × 10^−4^ a	2.31 × 10^−4^ a	1.77 × 10^−4^ b	2.38 × 10^−4^ a	*	**	ns
Chitin Degradation	1.08 × 10^−4^ a	1.06 × 10^−4^ a	1.11 × 10^−4^ a	9.80 × 10^−5^ a	ns	ns	ns
Sugar Utilization	1.70 × 10^−3^ a	1.48 × 10^−3^ a	1.61 × 10^−3^ a	1.57 × 10^−3^ a	ns	ns	ns
Sugar Transporters	6.66 × 10^−3^ a	5.46 × 10^−3^ b	6.29 × 10^−3^ ab	5.91 × 10^−3^ ab	ns	*	ns

Note: different letters represent significant differences among treatments (*p* < 0.05). ** *p* < 0.01; * *p* < 0.05; ns: There was no significant difference (*p* > 0.05).

## Data Availability

Data is contained within the article and Supplementary Material.

## Supplementary Materials

The following supporting information can be downloaded at: https://www.mdpi.com/article/10.3390/ijms251910790/s1.
