# Supplementary material for: Unveiling the Hidden Responses: Metagenomic Insights into Dwarf Bamboo (Fargesia denudata) Rhizosphere under Drought and Nitrogen Challenges
Source: Int J Mol Sci. 2024 Oct 8;25(19):10790. doi: 10.3390/ijms251910790 (PMC11477272; doi:10.3390/ijms251910790)
Supplement: Supplementary file 1 [file ijms-25-10790-s001.zip › ijms-3195260-supplementary.pdf]

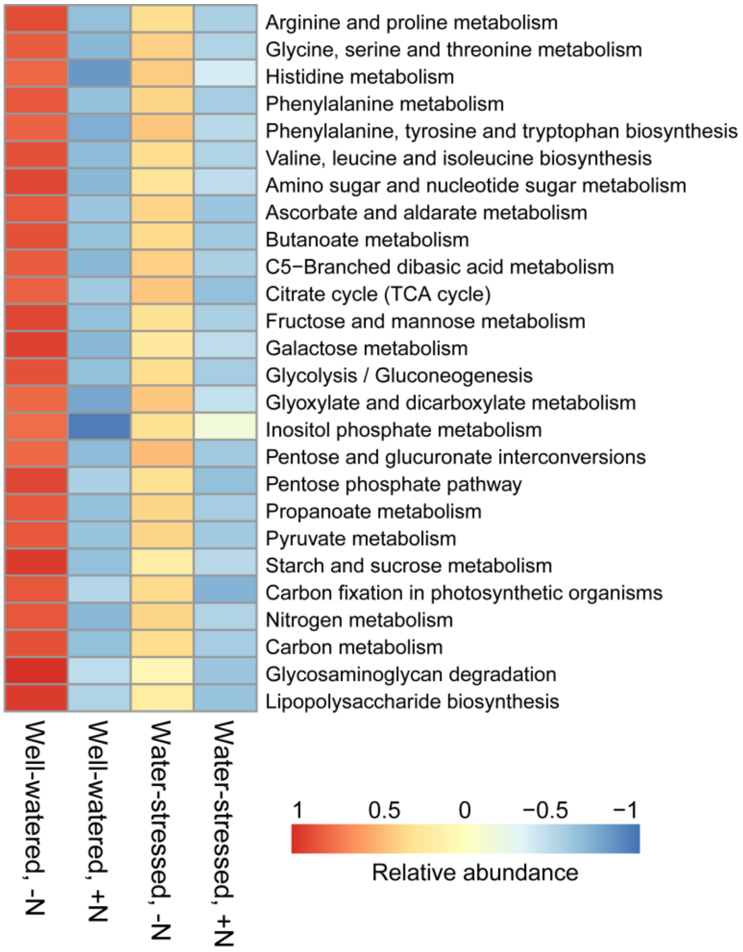

Figure. S1 KEGG pathway level-3 enrichment analysis

Table S1 Relative abundance of microbial species at the phylum level in rhizosphere soil of *Fargesia denudata* under different treatments

Phylum	Well-watered		Water-stressed		W	N	W×N
	-N	+N	-N	+N			
Acidobacteria	11.02%±1.24a	10.44%±0.32a	10.84%±0.64a	10.55%±1.51a	ns	ns	ns
Actinobacteria	11.3%±1.41b	17.35%±0.63a	15.95%±2.22b	16.36%±1.2ab	ns	ns	ns
Bacteroidetes	1.19%±0.12a	1.23%±0.05a	1.23%±0.08a	1.17%±0.15a	ns	ns	ns
Chloroflexi	2.38%±0.32a	2.48%±0.04a	2.58%±0.16a	2.69%±0.27a	ns	ns	ns
Cyanobacteria	1.63%±0.23a	1.44%±0.02a	1.47%±0.11a	1.49%±0.2a	ns	ns	ns
Firmicutes	2.36%±0.29a	2.18%±0.03a	2.24%±0.17a	2.27%±0.27a	ns	ns	ns
Gemmatimonadetes	2.02%±0.32a	1.86%±0.02a	1.89%±0.15a	1.76%±0.24a	ns	ns	ns
Nitrospirae	1.69%±2.69a	1.68%±0.33a	1.53%±3.33a	1.45%±3.26a	ns	ns	ns
Planctomycetes	1.66%±0.23a	1.59%±0.02a	1.59%±0.1a	1.57%±0.2a	ns	ns	ns
Proteobacteria	46.83%±0.04a	43.61%±0.28a	43.41%±0.17a	44.12%±0.17a	ns	ns	ns
Verrucomicrobia	3.65%±0.06a	3.3%±0a	3.23%±0.03a	3.65%±0.04a	ns	ns	ns
Euryarchaeota	0.4%±0.21a	0.36%±0.02a	0.37%±0.14a	0.37%±0.23a	ns	ns	ns
Deinococcus Thermus	0.33%±0.05a	0.31%±0a	0.32%±0.02a	0.32%±0.04a	ns	ns	ns
Armatimonadetes	3.43%±0.02a	3.24%±0a	3.29%±0.01a	3.35%±0.02a	ns	ns	ns
Chlorobi	9.64%±0.04a	6.6%±0a	7.49%±0.02a	7.72%±0.03a	ns	ns	ns
others	0.45%±6.13a	2.34%±0.29a	2.57%±4.51a	1.15%±4.35a	ns	ns	ns

Note: different letters represent significant differences among different treatments ($P < 0.05$)*** $P < 0.001$; ** $P < 0.01$; * $P < 0.05$; ns; There was no significant difference ($P > 0.05$).

Table S2 Effects of Nitrogen (N) Deposition and Drought Stress on microbial communities based on PERMANOVA analysis

	At the phylum level			At the genus level		
	F	R^2	*P*	*F*	R^2	*P*
Drought stress	0.134	0.013	0.897	1.010	0.917	0.265
Nitrogen deposition	0.821	0.076	0.445	1.169	0.105	0.006
Drought stress × Nitrogen deposition	0.748	0.219	0.602	1.063	0.285	0.024

Table S3 Relative abundance of microbial species at the genus level in rhizosphere soil of *Fargesia denudata* under different treatments

Genus	Well-watered		Water-stressed		W	N	W×N
	-N	+N	-N	+N			
Caballeronia	26.9%±0.2a	26.07%±0.38a	26.6%±0.29a	26.09%±0.73a	ns	ns	ns
Bradyrhizobium	6.44%±0.08ab	6.41%±0.18b	6.22%±0.13b	6.73%±0.14a	ns	*	*
Rhodoplanes	1.96%±0.06a	2.08%±0.11ab	1.86%±0.03b	2.04%±0.1a	ns	ns	ns
Mycobacterium	1.63%±0.07b	2.89%±0.13a	2.26%±0.31a	2.48%±0.52a	ns	ns	ns
Nitrospira	1.21%±0.01ab	1.16%±0.01a	1.17%±0.05ab	1.07%±0.06ab	ns	*	ns
Gemmatimonas	1.37%±0.04a	1.43%±0.01a	1.26%±0.05bc	1.16%±0.07c	**	ns	ns
Chthoniobacter	1.01%±0.03a	1.1%±0.12ab	0.96%±0.01ab	1.15%±0.05b	ns	ns	ns
Streptomyces	1.13%±0.01a	1.36%±0.03a	1.33%±0.1a	1.38%±0.12a	ns	ns	ns
Terrimicrobium	1.06%±0.06a	2.38%±0.13a	2.13%±0.53a	2.1%±0.59a	ns	ns	ns
Solirubrobacter	0.84%±0.04a	0.72%±0.05a	0.69%±0.04b	0.77%±0.03a	ns	ns	ns
Pseudolabrys	1.05%±0.04a	0.78%±0.09a	0.82%±0.1ab	0.95%±0.1ab	ns	ns	ns
Burkholderia	0.9%±0.03b	1.28%±0.04ab	1.22%±0.14a	1.27%±0.16ab	ns	ns	*
Gaiella	1.2%±0.01a	1.08%±0.01a	1.16%±0.06b	1.12%±0.05a	ns	ns	ns
Pyrinomonas	0.72%±0.01a	0.63%±0.04a	0.67%±0.04ab	0.63%±0.05a	ns	ns	ns
Dongia	0.58%±0.03ab	0.48%±0.02b	0.60%±0.10ab	0.63%±0.09a	ns	ns	ns
others	52.02%±0.28a	50.16%±0.28b	51.06%±0.84ab	50.44%±1.03b	ns	*	ns

Note: different letters represent significant differences among different treatments ($P < 0.05$)*** $P < 0.001$; ** $P < 0.01$; * $P < 0.05$; ns; There was no significant difference ($P > 0.05$)
